# Supplementary material for: Brain state and cortical layer-specific mechanisms underlying perception at threshold
Source: eLife. 2024 Nov 18;12:RP91722. doi: 10.7554/eLife.91722 (PMC11573349; doi:10.7554/eLife.91722)
Supplement: Supplementary file 1. — (a) Corresponding null hypothesis testing results. Null hypothesis testing results corresponding to the estimation statistics-based analyses. (b) Generalized linear model (GLM) coefficient values. Coefficients and significance values of the variables used in the GLM analysis. (c) GLM summary. Additional summary statistics of the GLM analysis. [file elife-91722-supp1.docx]

# Supplementary File 1a: Corresponding Null-Hypothesis Testing Results

| Result (Figure) | Null-Hypothesis Test | P-Value (Both monkeys) | P-Value (Monkey A) | P-Value (Monkey C) |
| --- | --- | --- | --- | --- |
|  | | | | |
| Pupil diameter (2B) | *t-*test (unpaired) | $p=6.63624e-09$ | $p=1.46705e-11$ | $p=0.000477729$ |
|  | | | | |
| Microsaccades (2D) | *X^2^-*test | $p\ll0.0001$ | $p\ll0.0001$ | $p= 3.085882e-02$ |
|  | | | | |
| Decoding performance [narrow-spiking] (3A) | *t-*test (unpaired)  Bonferoni Corrected for 3 comparisons, $=0.0166$ | Superficial:$p=1.438522e-41$ | Superficial:$p=9.114535e-12$ | Superficial:$p=4.86324e-39$ |
|  |  | Input: $p=8.589733e-45$ | Input: $p=7.091072e-48$ | Input: $p=0.2236990$ |
|  |  | Deep: $p=1.655900e-39$ | Deep: $p=7.422972e-31$ | Deep: $p=3.962452e-39$ |
| Decoding performance [broad-spiking] (3B) | *t-*test (unpaired)  Bonferoni Corrected for 3 comparisons, $=0.0166$ | Superficial:$p=4.636260e-25$ | Superficial:$p=3.171338e-13$ | Superficial:$p=1.70382e-32$ |
|  |  | Input: $p=0.2697764$ | Input: $p=0.1658690$ | Input: $p=9.269061e-20$ |
|  |  | Deep: $p=$ 2.755037e-35 | Deep: $p=5.577542e-39$ | Deep: $p=8.339443e-14$ |
|  | | | | |
| Target-evoked firing rate [single and multi-units] (3E) | *t-*test (paired)  Bonferoni Corrected for 3 comparisons, $=0.0166$ | Superficial: $p=4.19108e-05$ | Superficial: $p=0.0562592$ | Superficial: $p=0.00174249$ |
|  |  | Input: $p=1.10838e-11$ | Input: $p=0.00072401$ | Input: $p=0.000133564$ |
|  |  | Deep: $p=1.75826e-11$ | Deep: $p=0.00854264$ | Deep: $p=2.32077e-05$ |
| Target-evoked firing rate [broad-spiking] (3F) | *t-*test (paired)  Bonferoni Corrected for 3 comparisons, $=0.0166$ | Superficial: $p=0.0526902$ | Superficial: $p=0.0253932$ | Superficial: $p=0.28285$ |
|  |  | Input: $p=0.000124947$ | Input: $p=6.19734e-05$ | Input: $p=0.0122162$ |
|  |  | Deep: $p=0.00119012$ | Deep: $p=0.00430587$ | Deep: $p=0.0594886$ |
| Target-evoked firing rate [narrow-spiking] (3F) | *t-*test (paired)  Bonferoni Corrected for 3 comparisons, $=0.0166$ | Superficial: $p=0.103689$ | Superficial: $p=0.321259$ | Superficial: $p=0.226899$ |
|  |  | Input: $p=0.00570757$ | Input: $p=0.0317082$ | Input: $p=0.094339$ |
|  |  | Deep: $p=0.00393437$ | Deep: $p=0.423418$ | Deep: $p=0.00790649$ |
|  | | | | |
| Fano Factor modulation index [broad] (4B) | *t-*test (unpaired)  Bonferoni Corrected for 3 comparisons, $=0.0166$ | Superficial: $p=0.0102155$ | Superficial: $p=0.0258056$ | Superficial: $p=0.150999$ |
|  |  | Input: $p=0.418197$ | Input: $p=0.363723$ | Input: $p=0.889923$ |
|  |  | Deep: $p=0.708115$ | Deep: $p=0.507158$ | Deep: $p=0.570777$ |
| Fano factor modulation index [narrow] (S4) | *t-*test (unpaired)  Bonferoni Corrected for 3 comparisons, $=0.0166$ | Superficial: $p=0.676039$ | Superficial: $p=0.931029$ | Superficial: $p=0.626767$ |
|  |  | Input: $p=0.10532$ | Input: $p=0.435288$ | Input: $p=0.164457$ |
|  |  | Deep: $p=0.950487$ | Deep: $p=0.508305$ | Deep: $p=0.851834$ |
|  | | | | |
| Deep layer PPC (5B) | *t-*test (paired)  Bonferoni Corrected for 3 comparisons, $=0.0166$ | 3-12 Hz: $p=0.0142$ | 3-12 Hz: $p=0.0015$ | 3-12 Hz: $p=0.7400$ |
|  |  | 15-25 Hz: $p=0.4064$ | 15-25 Hz: $p=0.4875$ | 15-25 Hz: $p=0.6515$ |
|  |  | 30-80 Hz: $p=0.3600$ | 30-80 Hz: $p=0.5322$ | 30-80 Hz: $p=0.1052$ |
| CCA [pre-stimulus, S = superficial, I = input, D = deep] (6C, left) | *t-test (paired)*  Bonferoni Corrected for 3 comparisons, $=0.0166$ | I to S: $p=0.0069$ | I to S: $p=0.0166$ | I to S: $p=0.2329$ |
|  |  | I to D: $p=0.0287$ | I to D: $p=0.0141$ | I to D: $p=0.9494$ |
|  |  | S to D: $p=0.0359$ | S to D: $p=0.0107$ | S to D: $p=0.9821$ |
| CCA [stimulus, S = superficial, I = input, D = deep] (6C, right) | *t-test (paired)*  Bonferoni Corrected for 3 comparisons, $=0.0166$ | I to S: $p=0.0021$ | I to S: $p=0.0031$ | I to S: $p=0.3121$ |
|  |  | I to D: $p=0.0003$ | I to D: $p=0.0001$ | I to D: $p=0.4667$ |
|  |  | S to D: $p=0.0051$ | S to D: $p=0.0012$ | S to D: $p=0.6522$ |
|  | | | | |
| SSC modulation index [superficial-input] (7B, top) | *t-*test  Bonferoni Corrected for 3 comparisons, $=0.0166$ | 3-12 Hz: $p=0.925338$ | 3-12 Hz: $p=0.492935$ | 3-12 Hz: $p=0.324414$ |
|  |  | 15-25 Hz: $p=0.107304$ | 15-25 Hz: $p=0.47866$ | 15-25 Hz: $p=0.0818655$ |
|  |  | 30-80 Hz: $p=0.00394525$ | 30-80 Hz: $p=0.0337678$ | 30-80 Hz: $p=0.0714013$ |
| SSC modulation index [superficial-deep] (7B, middle) | *t-*test  Bonferoni Corrected for 3 comparisons, $=0.0166$ | 3-12 Hz: $p=0.0125919$ | 3-12 Hz: $p=0.196645$ | 3-12 Hz: $p=0.0163812$ |
|  |  | 15-25 Hz: $p=0.00116552$ | 15-25 Hz: $p=0.0035436$ | 15-25 Hz: $p=0.113136$ |
|  |  | 30-80 Hz: $p=0.00142568$ | 30-80 Hz: $p=0.00111326$ | 30-80 Hz: $p=0.192337$ |
| SSC modulation index [input-deep] (7B, bottom) | *t-*test  Bonferoni Corrected for 3 comparisons, $=0.0166$ | 3-12 Hz: $p=0.260698$ | 3-12 Hz: $p=0.0790771$ | 3-12 Hz: $p=0.628428$ |
|  |  | 15-25 Hz: $p=0.0358178$ | 15-25 Hz: $p=0.00766517$ | 15-25 Hz: $p=0.705241$ |
|  |  | 30-80 Hz: $p=0.0325516$ | 30-80 Hz: $p=0.00723361$ | 30-80 Hz: $p=0.72298$ |
| SSC modulation index [superficial-input] (7D, top) | *t-*test  Bonferoni Corrected for 3 comparisons, $=0.0166$ | 3-12 Hz: $p=0.00344279$ | 3-12 Hz: $p=0.00972663$ | 3-12 Hz: $p=0.1644$ |
|  |  | 15-25 Hz: $p=3.17111e-05$ | 15-25 Hz: $p=0.000184256$ | 15-25 Hz: $p=0.0559084$ |
|  |  | 30-80 Hz: $p=0.00396824$ | 30-80 Hz: $p=0.0243733$ | 30-80 Hz: $p=0.0964699$ |
| SSC modulation index [superficial-deep] (7D, middle) | *t-*test  Bonferoni Corrected for 3 comparisons, $=0.0166$ | 3-12 Hz: $p=0.0164846$ | 3-12 Hz: $p=0.034922$ | 3-12 Hz: $p=0.293683$ |
|  |  | 15-25 Hz: $p=0.0890114$ | 15-25 Hz: $p=0.0243132$ | 15-25 Hz: $p=0.956535$ |
|  |  | 30-80 Hz: $p=0.00441928$ | 30-80 Hz: $p=0.050113$ | 30-80 Hz: $p=0.0504199$ |
| SSC modulation index [input-deep] (7D, bottom) | *t-*test  Bonferoni Corrected for 3 comparisons, $=0.0166$ | 3-12 Hz: $p=0.241036$ | 3-12 Hz: $p=0.501995$ | 3-12 Hz: $p=0.340605$ |
|  |  | 15-25 Hz: $p=0.00140176$ | 15-25 Hz: $p=0.000982425$ | 15-25 Hz: $p=0.388365$ |
|  |  | 30-80 Hz: $p=6.84271e-05$ | 30-80 Hz: $p=0.000141679$ | 30-80 Hz: $p=0.149453$ |
|  | | | | |
| Superficial layer PPC (5-supplement 1B) | *t-*test (paired)  Bonferoni Corrected for 3 comparisons, $=0.0166$ | 3-12 Hz: $p=0.8307$ | 3-12 Hz: $p=0.6385$ | 3-12 Hz: $p=0.6207$ |
|  |  | 15-25 Hz: $p=0.2812$ | 15-25 Hz: $p=0.1918$ | 15-25 Hz: $p=0.8869$ |
|  |  | 30-80 Hz: $p=0.0037$* | 30-80 Hz: $p=0.0672$ | 30-80 Hz: $p=0.0290$ |
|  | | | | |
| Input layer PPC (5-supplement 1C) | *t-*test (paired)  Bonferoni Corrected for 3 comparisons, $=0.0166$ | 3-12 Hz: $p=0.3440$ | 3-12 Hz: $p=0.2469$ | 3-12 Hz: $p=0.1651$ |
|  |  | 15-25 Hz: $p=0.2517$ | 15-25 Hz: $p=0.2806$ | 15-25 Hz: $p=0.7294$ |
|  |  | 30-80 Hz: $p=0.1881$ | 30-80 Hz: $p=0.1045$ | 30-80 Hz: $p=0.1725$ |

* Although this p-value is significant, the PPC in both conditions is below 0, indicating there is no phase-locking in either condition (Vinck et al., 2010).

# Supplementary File 1b: GLM Coefficient Values

| Variable (Z-Scored) | Estimated Coefficient | P-Value |
| --- | --- | --- |
| Pupil Diameter | 0.11754 | $p=0.32869$ |
| Pretarget Microsaccades | -1.3116 | $p=6.0757e-08$ |
| Target Superficial FR | 0.22414 | $p=0.091946$ |
| Target Input FR | 0.3276 | $p=0.020068$ |
| Target Deep FR | 0.11399 | $p=0.45762$ |

# Supplementary File 1c: GLM Summary

|  | Value |
| --- | --- |
| Degrees of Freedom | 303 |
| Deviance (Full model) | 362.8493 |
| Deviance (Excluding microsaccades) | 426.5172 |
| Chi^2^-statistic (Full model) vs. constant model | 65.5 |
| Chi^2^-statistic (Full model) vs. constant model p-value | $p=8.88e-13$ |
